# Growth, immunity, and antioxidant activity responses of phytobiotics and probiotics incorporated into *Oreochromis niloticus* diets under stressors of unchanged water

**DOI:** 10.1038/s41598-025-30248-2

**Published:** 2025-12-29

**Authors:** Mohamed F. Abdel-Aziz, Rabab M. Alkaradawe, Hamed H. E. Saleh, Fatma Ragab Abouel Azm, Mohamed A. Elokaby, Abdel-Moneim M. Yones, Dalia S. Hamza, Naglaa R. A. Kasem

**Affiliations:** 1 Department of Aquaculture and Biotechnology, Faculty of Aquaculture and Marine Fisheries, Arish University, Arish, Egypt; 2 Zoology Department, Faculty of Science, Arish University, Arish, Egypt; 3 National Institute of Oceanography and Fisheries (NIOF), PO Box 11516, Cairo, Egypt; 4 Animal Nutrition and Clinical Nutrition Department, Faculty of Veterinary Medicine, Benha University, Toukh, 13736 Egypt; 5 Biology Department, College of Science, Jouf University, Sakaka, 72341 Saudi Arabia; 6 Department of Zoology, Faculty of Science, Benha University, Benha, 13518 Egypt; 7 Department of Zoology, Faculty of Science, Qena University, Qena, 83523 Egypt

**Keywords:** Growth performance, Nile tilapia, Hematology, Immunological, Antioxidant enzyme, Phytobiotic/probiotics, Ecology, Ecology, Physiology, Zoology

## Abstract

The purpose of this study was to examine the impacts of incorporating commercial mixture phytobiotic/probiotics as feed additives in terms of performance, survival rate proximate composition, hematological parameters, immunological response and antioxidant enzymes activity of *Oreochromis niloticus* reared under un-exchanged water system. Nile tilapia with an average beginning weight ranged from 48.49 to 52.50 g and were distributed in concrete ponds (1 m × 1 m × 1 m; L × W × H) at 20 fish / pond. Four treatments were performed as follows: control group (CG) fish fed a basal feed without water exchange and three groups were reared under zero water exchange with adding three different doses of commercial phytobiotic /probiotics on the basal feed (Garlex^®^, Superimmune^®^, and Gallipro 200^®^): Group 1: (200 mg, 500 mg, and 200 mg)/kg diet, Group 2: (400 mg, 1000 mg, and 400 mg)/ kg diet and Group 3: (600 mg, 1500 mg, and 600 mg)/ kg diet. This trial lasted for ninety days. Groups 1 and 2 had the best growth indices and survival rates, and there was not a significant (*P* ≤ *0.05*) distinction between them. Fish fed Groups 1 and 2 showed the greatest improvement in proximate body composition, immunological responses, and antioxidant enzyme activity, including lysozyme, superoxide dismutase (SOD), catalase (CAT), and glutathione (GSH). This study has detected that feed additives of commercial mixture of 200 mg/kg Garlex®, 400 mg/kg Super Immune®, and 200 mg/kg GallPro-200® led to improved growth and physiological status of *O. niloticus* under un-exchanged water ponds.

## Introduction

 Recently, Egypt’s aquaculture industry has had tremendous success, ranking it first among African nations and sixth globally^1^. Egypt’s aquaculture represents more than 80.5% of total seafood production, and its superiority in tilapia production has represented more than 88% of aquaculture production^2^ in the past decade. Nevertheless, the issue of satisfying the need for animal protein has not been adequately addressed. Rather, there is a continuous increase in the price of farmed fish^3^. The lack of high-quality fresh water is the biggest challenge that limits the expansion of Egyptian aquaculture projects^4^. Whereas, water problems arise from irrigation drainage that is influenced by agricultural seasons, variations in water levels all year, and that may be polluted with agricultural pesticides^5^. Therefore, the solutions proposed to solve this problem are to move from traditional farming systems (semi-intensive and intensive systems) to modern farming systems. But the inputs of modern farming systems, whether fixed costs (tanks, pumps, filters, and ventilation devices) or variable costs (feed or energy), are very expensive. Additionally, managing modern aquaculture systems requires a lot of experience^6^.

The traditional system in Egypt, particularly intensive systems, depends on water that changes^7,8^. Therefore, the high cost of energy required to run water pumps can occasionally result in a lack of water, a decline in the quality of the water, or an increase in production expenses for the owners of these farms. Water entering and exiting through pumps is essential for some crops. Accordingly, recommending prolonging water change periods through the use of immunostimulants without compromising growth rates is an appropriate solution to confront irrigation water shortages during a certain period, to reduce energy consumption, or to stop changing water due to its low quality of its source.

An immunostimulant is generally understood to be any substance, medication, stressor, or activity that directly interacts with and activates system cells to increase the innate or non-specific immune response. According to^9^ immunostimulants can be classified as chemical agents, bacterial preparations, polysaccharides, plant or animal extracts, dietary factors, and cytokines. Probiotics help by increasing feed consumption efficiency and by stimulating immune cells, which lowers the rates of pathogen infestation. Prebiotics may benefit by affecting the balance of advantageous gut flora and inducing the release of vital digestion enzymes, which increases fish’s availability of nutrients. Similarly, synbiotics improve survival rates and alter the microbial composition of the gastrointestinal system more effectively than probiotics or prebiotics alone, which raises aquaculture productivity^10^. Several studies confirmed the positive role for immunostimulants to improve immune functions of fish that reared under optimum conditions such as *Oreochromis niloticus*^11^ *Micropterus salmoides*^12^ *Cyprinus carpio*, *Acipenser baerii Brandt*^13,14^. However, only a small number of researches have assessed the impact of immune stimulants in stressful conditions, such as rainbow trout (*Oncorhynchus mykiss*) and Nile tilapia (*Oreochromis niloticus*)^15,16^. Besides, medicinal plants are novel feed additives and are generally inexpensive, sustainable, and easily obtainable^17,18^. Many of them have shown demonstrable benefits for growth, immunoregulation, and antimicrobial capability in various aquatic animals^19^. According to earlier reports, several medicinal herbs have the ability to increase aquatic animals’ appetites and serve as feeding attractants by chemically influencing fish eating behavior, leading to improved feed utilization, and then reducing water pollution in culture systems^20^.

In the same context, many species, such as *Oreochromis niloticus*^19,21^, *Paralichthys olivaceus*^22^, and *Catla catla*^23^ showed their responses with beneficial effects on healthy growth of probiotic and herbal combinations, either feed or watery additives.

Interestingly garlic *Allium sativum* in feeds showed a significant feeding attraction activity for Japanese seabass and then growth and feed efficiency value of fish were significantly increased^24,25^. Similar results for garlic were also reported in many other aquatic animals’ goldfish *Carassius auratus*^26^, Nile tilapia *Oreaochromis nilotica*^27^, and *Litopenaeus vannamei*^28^.

The objective of this study was to reduce stressful condition on Nile tilapia by supplementing their diets with commercial phytobiotic/probiotic combinations and evaluating how their growth and physiological changes responded to an intense system with un-exchanged water.

## Materials and methods

### Location of the trial

For ninety days, this experiment was conducted at the National Institute of Oceanography and Fisheries (NIOF), Fayoum, Egypt, in the Fish Nutrition Laboratory and Fish Research Station.

### Fishes, experimental groups

A 240 Nile tilapia *Oreaochromis niloticus* juvenile with an average initially weight of 50.27 g ± 0.88 (SE) was brought from a commercial farm in Fayoum, Egypt. Fish quickly transported in oxygenated water using tank (1m^3^) at Fish Nutrition Lab. and acclimatized for 10 days. After that fish were randomly distributed in 12 indoor concrete ponds (1 m × 1 m × 1 m; L × W × H) containing well-aerated water through air stone diffusers. Fish were stocked at rate of 20 fish/pond. Four groups were performed as the follows: control group (CG) fish fed a basal diet without water exchange and three groups were reared under zero water exchange with adding three various concentrations of commercial phyto/probiotics on the basal feed (Garlex^®^, superimmune^®^, and Gallipro 200^®^): Group 1: (200 mg, 500 mg, and 200 mg)/kg diet, Group 2: (400 mg, 1000 mg, and 400 mg)/kg diet and Group 3: (600 mg, 1500 mg, and 600 mg)/kg diet. Treatments were applied in triplicate.

### Dietary supplements and Preparation of a tested diets

The ingredients of the basal feed were formulated to contain 30% crude protein, it was milled and well mixed without the supplementary diet for control groups and with adding dietary supplements (Garlex^®^, Superimmune^®^, and Gallipro 200^®^) for the treatments Group 1, Group 2 and Group 3 (Table, 1). The mixture was piloted by a pellet mill with a 3 mm die. Pellets were dried at 30 ˚C in an oven and stored at 5 ˚C in Refrigerator.

### Water quality indicators

The water physiochemical indicators were monitored, statistically analyzed, and recorded in Table 2. Water temperature, pH, and oxygen dissolved (DO) were measured daily by an Orion digital pH meter and an oxygen meter (Cole Parmer model 5946). Ammonia, and nitrite were measured every week by chemical methodology^29^, and unionized ammonia was calculated by the relationship between temperature and pH according to the data of^30^.

**Table 1:** Formulation and chemical analysis of the experimental diets (% DM basis).

Ingredients g/100 g	Groups					
	CG	Group 1	Group 2		Group 3	
Fish meal	16	16	16		16	
Soy bean meal	35.00	35.00	35.00		35.00	
Yellow corn	32.80	32.80	32.80		32.80	
Wheat bran	12	12	12		12	
Starch	1	1	1		1	
Soy oil	3	3	3		3	
^1^Vitamin & mineral	0.2	0.2	0.2		0.2	
Total	100	100	100		100	
Supplementary diets mg/kg						
^2^Garlex^®^	-	200	400		600	
^3^Superimmune^®^	-	500	1000		1500	
^4^GallPro-200^®^	-	200	400		600	
Chemical analysis of the basal diet on basis dry matter, %						
Dry matter	91.06					
Crude protein	29.82					
Ether extract	6.8					
Crude fiber	4.10					
Ash	5.80					
^5^Nitrogen free extract (NFE)	53.48					
^6^ Gross energy kcal/g	4.48					

1-Super mix: Vitamins and minerals mixture each 1Kg of mixture content: 62.5 m I.U. vit A, 25 m I.U. vit D3, 25 g vit E, 1.75 g vit K, 0.5 g vit. B1, 2.75 g vit. B2, 1.25 g vit. B6,10 mg.vit. B12, 20 g.niacin, 500 mg.Folic acid, 50 mg.Biotin, 37 g Zinc, 22 g. Iron, 31 g.Manganese, 2.5 g Copper, 50 mg.Coblat, 113 mg Selenium, 650 mg iodine. Neofarma, Italy.

2-Garlex Garlic mix, Hebei Kangdali pharmaceutical company, China.

3-Superimmune^®^ Powder Biological Antimycotoxins And Growth Promoter for Poultry. Composition/kg Purified yeastcell wall extract (*Saccharomyces cerevisiae*) 980 g, Beta-glucans 240 gm, Mannans 180 g, silicate 10 g and carrier of calcium carbonate up to 1 kg. Neofarma, Italy.

4- GallPro-200^®^ growth promoter probiotics: Additive/kg.

*Bacillus subtillis* spore concentrate (DSM 17299), No of spores min.4 × 1012 CFU/kg of product, Sodium aluminon silicate 10 g and carrier of calcium carbonate up to 1 kg. Biochem zusaotzstoffe Handlels-und Produktionsgesellschaft mbH (Küstermeyerstraße, Germany).

5-NFE was calculated by difference.

6-Gross energy was calculated according (NRC, 1993).

CG: control was fed a non-supplemented basal diet, at the high stocking (20 fish per 1 m3), and completely unexchanged water with 0% water exchange.

Group 1: fed a basal diet fortified with *(200 mg*,* 500 mg and 200 mg of Garlic mix*,* Super immune*,* and Gallipro 200)* kg^− 1^ diet at stocking (20 fish per 1 m^3^), with 0% water exchange.

Group 2: fed a basal diet fortified with (400 mg, 1000 mg and 400 mg of Garlic mix, Super immune, and Gallipro 200) kg^− 1^ diet at stocking (20 fish per 1 m^3^), with 0% water exchange.

Group3: fed a basal diet fortified with (600 mg, 1500 mg and 600 mg of Garlic mix, Super immune, and Gallipro 200) kg^− 1^ diet at stocking (20 fish per 1 m^3^), with 0% water exchange.

**Table 2:** Means (*n* = *3*) of water quality parameters during the experimental period.

Items	Groups				PSE*
	CG	Group 1	Group 2	Group 2	
Temperature, ˚ C	26.75	26.60	26.55	26.60	0.076
pH	8.90	8.87	8.94	9.07	0.117
Dissolved oxygen, mg/L	3.35	3.62	3.80	3.55	0.505
Unionized-Ammonia, mg/L	0.13	0.09	0.08	0.08	0.09
Nitrite, mg/L	1.81	1.70	1.69	1.81	0.12

*, Pooled standard error.

CG: control was fed a non-supplemented basal diet; Group 1: fed a basal diet fortified with (200 mg, 500 mg and 200 mg of Garlic mix, Superimmune, and Gallipro 200) kg^− 1^ diet; Group 2: fed a basal diet fortified with (400 mg, 1000 mg and 400 mg of Garlic mix, Super immune, and Gallipro 200) kg^− 1^ diet; Group3: fed a basal diet fortified with (600 mg, 1500 mg and 600 mg of Garlic mix, Superimmune, and Gallipro 200) kg^− 1^ diet.

### Biological indicators

Total weight gain (WG), average weight gain/day (ADG), Relative weight gain (RWG, %), specific growth rate (SGR), Feed conversion Efficiency (FCE), survival rate (SR), and condition index (CF).

These parameters were calculated according the following equations:

$${\text{WG, g}}\,{\mathrm{=}}\,{\text{final weight }}\left( {{\mathrm{W2}}} \right){\text{--initial weight }}\left( {{\mathrm{W1}}} \right)$$

ADG, g/day = average weight gain, g/experimental period, day,

$${\text{RWG, \% = [(W2--W1) / W1]}} \times 100$$

SGR, %/day = [(ln W2-ln W1)/days] × 100 whereas ln: is the natural log.

$${\text{FCE, \% = }}\left( {{\text{WG, g / Feed intake, g}}} \right) \times 100$$

$${\text{SR\% = }}\left( {{\text{Number of fishes at end/ Number of fishes at start}}} \right) \times {\mathrm{100}}$$

CF, % = (W2/L^3^) × 100 whereas L: is the final length of fish in cm.

### Sampling

At the finish of the trial, blood samples were taken at random from five fish each replication (15 fish per treatment). Clove oil was used to anesthetize fish at a concentration of 0.1 ml/l. Nine fish were used to sampling blood and six fish were euthanized by being frozen (−5 C) after being anesthetized, as previously mentioned for analyzing the body composition^31^.

#### Blood sampling

Blood was drawn from the caudal vein using a 3-ml syringe. Each blood sample was emptied in two micro tubes one of them contained EDTA to prevent coagulation and immediately examined for hematology analysis and other micro tubes did not contain EDTA to measure the serum parameters^30^.

##### Hematological and serum biochemical assay

Red blood cells (RBCs), White blood cell (WBCs), Hemoglobin (Hb) and Hematocrit (Hct) were analyzed using fully automatic hematological analyzer using commercial test kits (Bio-diagnostic, Egypt), serum biochemicals such as plasma aspartate aminotransferase (AST), alanine aminotransferase (ALT), serum glucose, total protein, urea, and creatinine were measured by calorimetric assay^32,33^.

##### Immunological-antioxidant indices

Commercial ELISA kits available from Biosource Inc. (San Diego, California, USA) were used to detected plasma lysozyme activity (Catalog No.: MBS725718), Immunoglobulin M (IgM; catalog No.: CSB-E07978r) and Complement C3 (MD1102094) were quantitatively determined using turbidimetric assay as described by^34,35^. Additionally, interleukin 10 (IL-10- catalog No. MBS269138)^36^, and Nitric oxide (NO) was estimated according to^37^. In the same manner calorimetric methods have been used to measure the amount of malondialdehyde. (MDA; catalog No.: MBS268427), superoxide dismutase (SOD; catalog No.: CSB-E08555r), catalase (CAT; catalog No.: MB2600683), and Glutathione (GSH: Catalog No. CSB-E12144r)^38^.

#### Body composition analysis

Fish carcasses and feeds were chemically analyzed in accordance with^39^. The coefficients 23.62, 39.5, and 17.56 KJ/g for CP, EE, and carbs, respectively, were used to estimate the gross energy (GE) of formulated diets^40^.

### Statistical analysis

Shapiro-test Wilk’s was undertaken to ensure that the collected data were normal and analyzed by one-way analysis of variance (ANOVA) at a 95% confidence limit, using SPSS software, version 16. Duncanʼs Multiple Range 23 test was employed to compare means when F-values from the ANOVA were significant (*P* < *0.05*).

## Results

### Water quality super

Table 2 presents the averages of the water physiochemical indices which ranged as follows: the water temperature (26.55–26.75 °C) pH levels (8.87–9.07), DO mg/L (3.35–3.80 mg/L), unionized ammonia (0.08–0.13 mg/L), and NO_2_ (1.69–1.80 mg/L). All this indicator *was* not significantly (*P* ≤ *0.05*) between all treatments.

### Fish growth performance

**Fig. 1:**
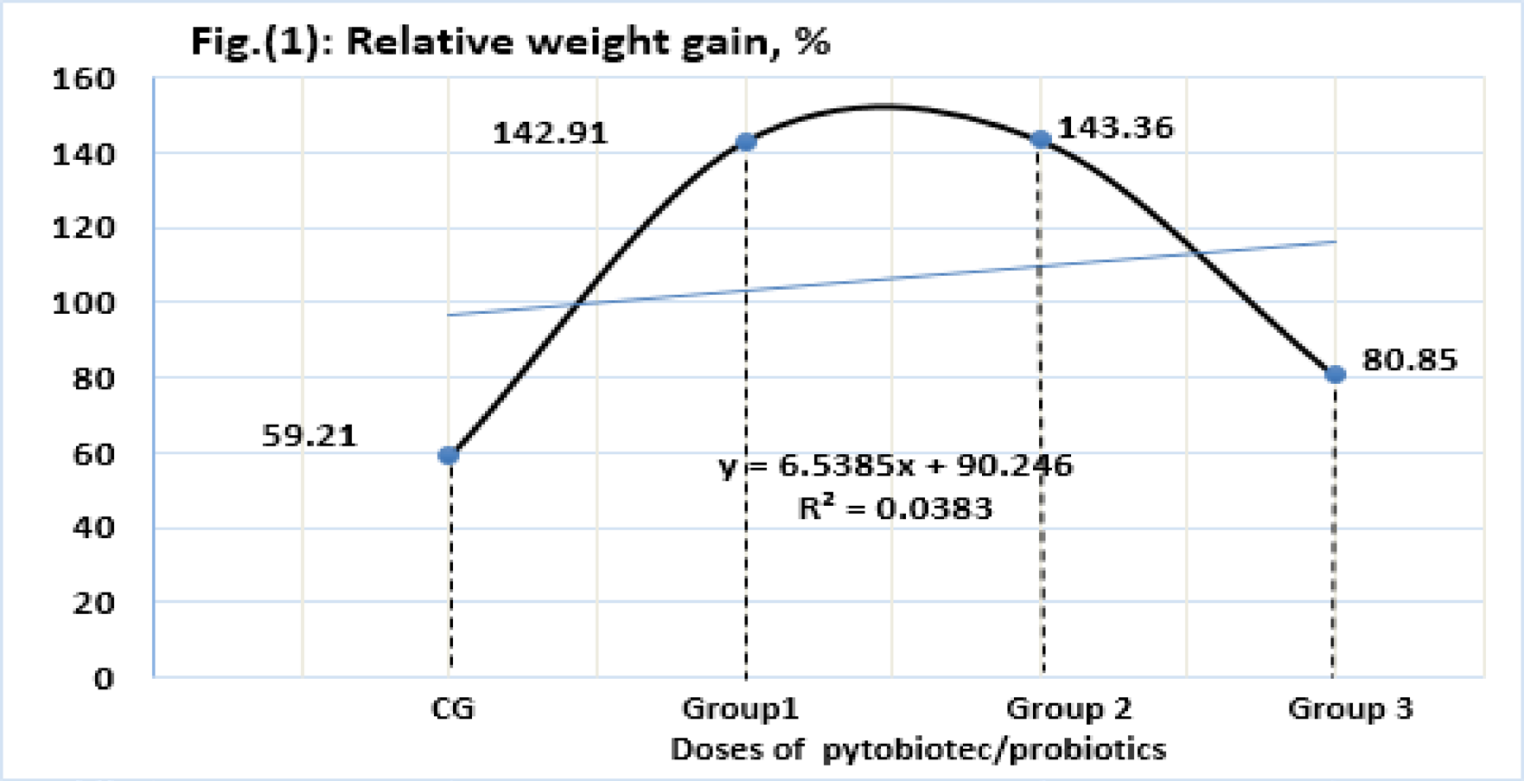
Weight gain % of Nile tilapia fed supplementary diet of phytobiotcs/probiotics under unchanged water for 90 days CG: control was fed a non-supplemented basal diet; Group 1, Group 2, and Group 3: fed a basal diet fortified with (200 mg, 500 mg, and 200 mg; 400 mg, 1000 mg, and 400 mg; 600 mg, 1500 mg, and 600 mg of Garlic mix, Superimmune, and Gallipro 200) kg-1 diet respectively.

Growth indices are shown in (Table 3) the statistical analysis appeared significant differences (*P* ≤ *0.05*) among all treatments with all parameters exclusion CF. Survival rate significantly varied in treatments. The growth, feeding utilization, and survival percentage of the treated groups that received a diet enriched with phytobiotics/probiotics were better than those of the control group. Group 2 exhibited the largest growth, with a weight gain percentage of 143.3; Group 1 and Group 3 had weight gain percentages of 142.9 and 80.85%, respectively (Fig. 1).

**Table 3:** Growth parameters and some body indices of nile tilapia fed supplementary diet of phytobiotcs/probiotics under unchanged water for 90 days.

Items	Groups				PSE*
	CG	Group 1	Group 2	Group 3	
W1, g	50.78	48.49	49.31	52.50	2.46
W2, g	80.86^c^	117.80^a^	120.00^a^	94.95^b^	12.49
WG, g	30.07^c^	69.30^a^	70.70^a^	42.45^b^	12.65
ADG, g/day	0.33^c^	0.77^a^	0.78^a^	0.47^b^	0.14
SGR, %/day	0.52^c^	0.98^a^	0.98^a^	0.63^b^	0.11
FCE, %	22.80^c^	65.22^a^	65.89^a^	33.11^b^	6.60
SR, %	55^c^	95^a^	95^a^	90^b^	10.00
CF, %	2.00	1.92	1.84	1.76	0.52

a, b, c, d, e in the same row with different superscripts are significantly different (*P* ≤ *0.05*, *n* = *3*).

*, Pooled standard error.

W1; initial weight; w2: final weight; WG: weight gain; ADG: average daily gain; SGR: specific growth rate, FCE: feed conversion efficiency; SR: survival rate; CF: condition factor.

CG: control was fed a non-supplemented basal diet; Group 1, Group 2, and Group 3: fed a basal diet fortified with (200 mg, 500 mg, and 200 mg; 400 mg, 1000 mg, and 400 mg; 600 mg, 1500 mg, and 600 mg of Garlic mix, Superimmune, and Gallipro 200) kg-1 diet respectively.

### Indicators of hematological and biochemical

Results of the blood hematology are displayed in (Table 4) and indicate a considerable variation among groups, where the highest values of WBC, Hb, RBC and Hct. were obtained with groups that administered dietary phytobiotics/probiotics especially Group2.

**Table 4:** Hematological parameters of nile tilapia fed supplementary diet of phytobiotcs/probiotics under unchanged water for 90 days.

Items	Groups				PSE*
	CG	Group 1	Group 2	Group 3	
RBCs (10^6^/mm^3^)	1.05^c^	1.27^b^	1.93^a^	1.18^bc^	0.12
WBCs (10^3^/mm^3^)	108.20^c^	110.60^bc^	118.50^a^	110.85^b^	2.47
Hb (g/dl)	5.29^c^	7.50^b^	8.40^a^	7.30^b^	0.45
Hct, %	18.90^b^	18.50^b^	23.0^a^	17.38^b^	1.50

a, b, c, d, e in the same row with different superscripts are significantly different (*P* ≤ *0.05*, *n* = *3*).

*, Pooled standard error.

RBCs: red blood cells; WBCs: white blood cells; Hb: hemoglobin; Hct: hematocrit.

CG: control was fed a non-supplemented basal diet; Group 1, Group 2, and Group 3: fed a basal diet fortified with (200 mg, 500 mg, and 200 mg; 400 mg, 1000 mg, and 400 mg; 600 mg, 1500 mg, and 600 mg of Garlic mix, Superimmune, and Gallipro 200) kg-1 diet respectively.

Blood biochemistry is presented in Table 5. Data showed noticeable differences (*P* < *0.05*) among treatments in creatinine, Urea, ALT, AST, glucose, and total protein. Treated-groups had the best indicators of serum assay compared to untreated group. Group 2 recorded the lowest values in all indicators except total protein had the highest from the other groups.

**Table 5:** Serum parameters of nile tilapia fed supplementary diet of phytobiotcs/probiotics under unchanged water for 90 days.

Items	Groups				PSE*
	CG	Group 1	Group 2	Group 3	
Creatinine, mg/dl	0.40^b^	0.52^a^	0.39^b^	0.46^ab^	0.03
Urea, mg/dl	9.15^a^	8.10^a^	7.61^ab^	6.78^ab^	0.47
Glucose, mg/d	82^a^	79^b^	70^c^	83^a^	2.40
ALT, U/l	10.10^a^	6.35^c^	6.38^c^	7.70^b^	0.50
AST, U/l	46.70^a^	32.55^b^	44.15^a^	47.35^a^	2.67
Total protein, g/dl	2.80^b^	2.74^b^	3.05^b^	3.01^b^	0.14

a, b, c, d, e in the same row with different superscripts are significantly different (*P* ≤ *0.05*, *n* = *3*).

*, Pooled standard error.

CG: control was fed a non-supplemented basal diet; Group 1, Group 2, and Group 3: fed a basal diet fortified with (200 mg, 500 mg, and 200 mg; 400 mg, 1000 mg, and 400 mg; 600 mg, 1500 mg, and 600 mg of Garlic mix, Superimmune, and Gallipro 200) kg-1 diet respectively.

**Table 6:** Immunological parameters and antioxidant enzymes activities of nile tilapia fed supplementary diet of phytobiotcs/probiotics under unchanged water for 90 days.

Items	Groups				PSE*
	CG	Group 1	Group 2	Group 3	
IgM (ng/ml)	110.75^c^	137.50^bc^	170.00^a^	1510^a^	20.11
C3 (mg/dl)	61.50	66.00	79.00	73.00	2.83
NO (u mol/l)	17.50^c^	25.00^ab^	34.00^a^	24.00^ab^	2.81
IL10 (Pg/ml)	188.00^c^	2070^bc^	280.00^a^	2210^b^	14.25
Lysozyme (ng/ml)	4.75^c^	8.25^b^	16.75^a^	7.00^b^	1.94
CAT (n mol/ml)	4.35^c^	6.80^b^	10.20^a^	6.45^bc^	0.87
SOD (ng/ml)	113.50^c^	150^b^	200.00^a^	142^bc^	12.89
MDA (n mol/ml)	13.15^a^	9.72^b^	4.25^c^	8.00^b^	1.16
GSH (ng/ml)	5.25^c^	10.50^b^	16.00^a^	9.00^b^	2.14

a, b, c, d, e in the same row with different superscripts are significantly different (*P* ≤ *0.05*, *n* = *3*).

*, Pooled standard error.

(IgM): Immunoglobulin M, (IgM): Immunoglobulin G, C3: complement, (IL-10): Interleukin 10, (NO) Nitric oxide, (CAT): Serum Catalase, (SOD): Superoxide Dismutase, (MDA): Malondialdehyde, (GSH): Glutathione.

CG: control was fed a non-supplemented basal diet; Group 1, Group 2, and Group 3: fed a basal diet fortified with (200 mg, 500 mg, and 200 mg; 400 mg, 1000 mg, and 400 mg; 600 mg, 1500 mg, and 600 mg of Garlic mix, Superimmune, and Gallipro 200) kg-1 diet respectively.

### Immunological indicators and antioxidant properties

Table (6) showed that treated-fish had considerably greater in values of lysozyme activity, Complement C3, interleukin 10, Nitric oxide, malondialdehyde, superoxide dismutase (SOD), catalase (CAT), and Glutathione (GSH) than control group. Where Group 2 was the highest in these parameters and recorded the lowest MDA.

### Chemical composition of carcass

At the end of trial duration, fish carcass was chemically analyzed, and their results presented in (Table 7). Carcass content of protein significantly changed between treatments, where the highest values were recorded with Group 1, 2 and 3 respectively in comparison with control. Group1 and 2 had the lowest fat content compared to group 3 and control. On the other side, Ash values are not significantly different between Groups 1,2 and 3, but they were considerably lower than control group.

**Table 7:** Chemical composition of fish carcass (On basis wet weight%) of nile tilapia fed supplementary diet of phytobiotcs/probiotics under unchanged water for 90 days.

Items	Groups				PSE*
	CG	Group 1	Group 2	Group 3	
Moisture, %	80.78	80.20	79.55	79.63	1.79
Crude protein, %	15.38^b^	16.35^a^	16.83^a^	16.72^a^	0.35
Fat, %	2.41^a^	2.28^b^	2.47^b^	2.55^a^	0.14
Ash, %	1.38^a^	1.154^b^	1.00^b^	1.08^b^	0.26

a, b, c, d in the same row with different superscripts are significantly different (*P* ≤ *0.05*, *n* = *3*).

*, Pooled standard error.

CG: control was fed a non-supplemented basal diet; Group 1, Group 2, and Group 3: fed a basal diet fortified with (200 mg, 500 mg, and 200 mg; 400 mg, 1000 mg, and 400 mg; 600 mg, 1500 mg, and 600 mg of Garlic mix, Superimmune, and Gallipro 200) kg-1 diet respectively.

## Discussion

Results in this experiment indicate that a commercial mixture of Phytobiotic/probiotics enhanced growth, survival percentage, carcass composition, blood indices, immunity, and antioxidant enzyme activities in *Oreochromis niloticus*, under an un-exchanged water system. Although Table 2 showed that water temperature, pH, DO, and mg/l were within acceptable limits for tilapia growth as recorded by^30,41^ all treatments deteriorated severely, especially unionized ammonia and nitrite. Whereas^42,43^ suggested that increased NH3 above 0.05 or 0.068 ppm has a negative effect on biological parameters^44^, found that red tilapia can die from exposure to unionized ammonia (NH3) at concentrations between 0.11 and 0.15 if prolonged. Another study by^45^ proposed that NH_3_ levels below 0.1 mg/l are safe for aquaculture. In our study, the means of NH3 ranged between 0.0855 and 0.125 ppm and led to decreased growth rate, with an increase in the mortality rate of the control group in comparison with the treated groups with photobiotic/probiotics. There is a role for aeration in reducing the severity of ammonia toxicity under unchanged water conditions, as was suggested by^46^. There are factors impacting water quality that are primarily related to fish waste and feed^47^. Therefore, it’s important to investigate certain nutritional alternatives in order to enhance fish feed consumption and reduce water fouling, in addition to improving growth performance. Additionally, aeration and natural immunostimulants (phytobiotic/probiotics) can help reduce the stress caused by exposure to high ammonia concentrations. In partial similarity with our study^48,49^ using the probiotics in recirculating aquaculture system (RAS) improved water quality, immune responses, and feed efficiency of culture species.

In the same trend, phytobiotics or medical plants help to decrease the stress of water pollution in culture systems through improved feeding utilization, immunoregulation, and antimicrobial capability in various aquatic animals^50^.

The commercial mixture of phytobiotic/probiotics supplements used in our study included garlic as a natural growth promoter, immunostimulant, and antioxidant. Also, commercial probiotics include purified yeast (*Saccharomyces cerevisiae*), Beta-glucans, Mannans, silicate, *and Bacillus subtilis*. By using probiotics, the fish’s ability to feed, ingestion, digestion, absorption, metabolism, immunological function, and health improved. Prebiotics may benefit by affecting the balance of advantageous gut flora and inducing the release of vital digestive enzymes, which increases the availability of nutrients for fish. Enzymes involved in digestion (carbohydrates, phosphatases, esterase, lipases, peptidases, cellulases, and proteases) are produced by gut microbes in fish, including some species commonly used as probiotics^51^. Increased synthesis of amylase, lipase, and protease was observed in the intestine of tilapia fed diets containing Bacillus subtilis and unknown “photosynthetic bacteria”^52^. According to^53,54^ probiotics can also be used in fish feeds to enhance the amount of high-cellulose carbohydrate sources as a result of increasing digestive enzyme activity. This tactic may assist fish overcome the low digestibility and anti-nutritional properties of plant-based substitute meal ingredients. Additionally, it had been found that probiotic treatment considerably enhanced the length and density of microvillus in the mid-intestine of tilapia^55^.

Besides, garlic supplements can boost immunity, stimulate appetite, encourage growth, and enhance the quality of fresh produce^56^. Allicin, a powerful flavor found in garlic, may increase appetite and facilitate better digestion. Also, allicin can enhance digestion by stimulating the intestinal flora and suppressing harmful bacteria, so enhancing the overall well-being of fish raised in culture^57^. Furthermore, by promoting the release of bile acids, garlic has been demonstrated to enhance the regulation of digestion^58^.

In the same context^59^, found that Japanese seabass fed a diet containing garlic oil boost had the best digestion efficiency. Emphasizing the importance of garlic, there are many biologically active substances found in garlic, such as sugars, amino acids, and their equivalents that are soluble in oil^60^.

Numerous studies have shown that *S. cerevisiae* and *Bacillus subtilis* impact the growth of a number of fish species, such as rainbow trout (*Oncorhynchus mykiss*)^61^, common carp (*Cyprinus carpio*n^62^, and Nile tilapia^63,64^.

In our study, dietary supplements (Garlex^®^, Superimmune^®^, and Gallipro 200^®^) significantly enhanced the growth (WG, SGR) and feed conversion efficiency (FCE) of *Oreochromis niloticus* compared to the control. Wherein, WG% after 90 days increased in Group 2 by 84.15% than un-treated group. Similarly^65^, reported an increase in the daily growth rate by 33% of *Oreochromis niloticus* fed on Biogen^®^ (a commercial product that contains *B. subtilis*). Also^25^, mentioned that dietary garlic greatly increased the feed efficiency of *Japanese seabass*, enabling them to satisfy body growth requirements and reduce the loss of nutrients into water, which in turn contributes to reducing pollution. In the current study, the decreased growth performance observed in Group 3, as the dosage of phytobiotics and probiotics increased, could have negatively affected the animals’ immune or physiological status, ultimately leading to a decline in growth performance. These findings are consistent with those of^66^ who reported that fish fed high concentrations of baker’s yeast exhibited slower growth. Another study showed that Rainbow trout had significantly lower growth when fed on a commercial probiotic containing *Saccharomyces cerevisiae* and *Saccharomyces elipsoedas* at 1.5% and above^61^. In the same vein^25^, found that garlic did not significantly increase the SGR and WG of Japanese seabass as compared with the control. Generally, it is difficult to draw concrete conclusions and provide specific recommendations on the effects of dietary probiotics or garlic on the growth performance of fish. Wherein, the studies vary widely about fish species, fish age and size, stocking density, diet composition, concentration of doses, feed allowances, feeding duration, and, of course, type and source of probiotic. Fish health, physiological and pathological conditions are measured using blood parameters, which are an effective and sensitive indicator^67^. Results of effects of the commercial mixture of phytobiotic/probiotics as feed supplementary feeds on hematological and serum biochemical of fish illustrated improvements in the physiological status of treated fish groups especially Group 2 which had higher WBCs, RBCs, Hb and Hct comparable to the control group. It is well-known that these features of cultured fish are commonly used to identify stress and disease^68^. The increasing WBCs may be a result of increased feed protein utilization, which in turn may have increased leucocyte synthesis in the kidney and spleen tissue, and they are a defense against infections. Fish with higher RBCs, Hb, and Hct levels are more active. The ability of blood to bind oxygen is directly correlated with increasing these markers^69,70^. Furthermore, treated groups had the best blood serum indices, especially Group 2, which recorded the lowest blood glucose, AST, ALT, and the highest total protein. It is believed that when fish are kept in stressful environments, their body physiology and metabolic activity rise. In order for fish to recuperate energy during stressful situations, a higher level of plasma glucose may be needed^25^. High glucose levels often signify increased stress in fish, and plasma glucose is considered a key stress indicator in these animals^71^. Increased levels of AST and ALT enzymes may suggest the degeneration and/or destruction of the liver. Additionally, according to^72^, these enzymes may be used to evaluate the toxicity of supplements and/or diets compared to the probiotic-free control group and increased levels of AST and ALT enzymes may suggest the degeneration and/or destruction of the liver. The probiotic-supplemented groups in the current study showed lower values of these parameters, indicating that probiotics produced optimal physiological function and improved liver health state in fish. Noteworthy that levels of total protein in blood are correlated with liver-synthesized protein, while elevated blood protein levels may be linked to a stronger innate immune response^73^.

Numerous studies agreed with our findings^74^. reported that garlic acts as a natural antibiotic, considering its allicin content, and has been shown to decrease blood pressure, cholesterol, and blood sugar. According to^69^ juvenile starlet sturgeon fed garlic extracts showed a substantial decrease in blood glucose levels. Furthermore^75^, discovered that garlic may enhance rainbow trout’s (*Oncorhynchus mykiss*) antioxidative status; however, the amount of garlic in the feed needs to be well adjusted. In the same positive track, dietary probiotics reduced plasma GOT (or aspartate aminotransferase, AST) and GPT (or alanine aminotransferase, ALT) levels, which are often used for the evaluation of liver enzymes^76^.

Notably and unmistakably diets supplemented with probiotics or phytobiotics have significant effects on stress tolerance capacity, immunity of fish and have a potential efficacy in diminishing oxidative stress among aquatic organisms, including fish and shellfsh^77,78^. In this study, several systemic, non-specific immune functions are enhanced by dietary Photobiotic/probiotic supplementation, including lysozyme activity, IgM, Complement C3, IL-10, and Nitric oxide (NO). lysozyme activity peripheral, blood immune cell counts, alternative complement activity, phagocytic ability of leucocytes, neutrophil migration and adherence, plasma bactericidal activity, respiratory burst, myeloperoxidase, and superoxide dismutase activities. According to^79^, there appears a correlation between elevated lysozyme levels in fish blood and phagocyte, or WBC, production. The ability of β-glucan to stimulate phagocytic cells to produce antimicrobial compounds, including lysosomal enzymes, the complement system, and reactive oxygen species (ROS), is commonly acknowledged and has played a vital role as a chemical signaling molecule within the organism. Also, treated Zebrafish with probiotics showed lower levels of oxidative stress, which may indicate improved hepatic stress tolerance^80^. Furthermore, fish blood has a high concentration of the primary systemic isotype, IgM, which is essential for pathogen neutralization and opsonization^81^.

Actually, antioxidant enzymes like SOD, CAT, and GSH protect fish cells against the harmful effects of several free radicals, including superoxide and hydrogen peroxide^82^. However, oxidative stress caused a number of fatty acids to be oxidized, which results in the production of malondialdehyde (MDA).

MDA and SOD have opposing antioxidant activity, and MDA is an indicator of lipid peroxidation and cell injury^83^. All immunological indices and antioxidant activity were higher in the treated groups, especially Group 2, rather than the control group, which had the highest MDA level, according to our data. Fish welfare can therefore be protected by a well-developed antioxidant system that guards against the oxidation of such fatty acids. Several studies reported that different dietary probiotics or phytobotics can increase lysozyme and improve the fish antioxidant system as well as ameliorate oxidative stress^84^. Similar effects have also been observed in several fish species that showed enhanced immunity by applying probiotic, prebiotic, and symbiotic feeding^85^. Major mechanisms of action of probiotics and phytopiotcs include enhancement of epithelial barrier function, improved adhesion to intestinal cells and pathogen inhibition by occupying adhesion sites, production of antibacterial substances, and regulation of the immune function^86^.

Carcass proximate analysis showed that fish fed a dietary phytobiotic/probiotics (Groups 1and 2) supplemented diets had significantly higher protein content and lower fat content compared with the other treatments. This increase in protein content could have resulted from increased nutrient deposition. This is consistent with studies on probiotic-fed *Oeochromis niloticus*^65,87,88^. However, other research has shown that probiotics had no influence on the amount of protein, fat, or ash^89^. According to^90^ there was no discernible impact of probiotics or herbs on the overall body composition of young fish. Similarly^91^, found that garlic extract supplementation had no effect on the whole-body amino acid composition of juvenile sterlet sturgeon (*Acipenser ruthenus*). Whatever, the production of fish with more protein and less fat is more suitable for consumers, as recorded by^64,92^.

## Conclusion

In general, feed additives of commercial mixture phytobiotic/probiotic at doses of 200 Garlex^®^, 400 Superimmune^®^, and 200 GallPro-200^®^ mg kg⁻¹ lead to improved fish growth performance, feed efficiency, and boosted immunological and antioxidant enzyme activities of *O. niloticus* under unexchanged water. Future research should focus on the sustainability and optimal use of water resources. In cases of fish culturing under unchanged water, the average water quality should be measured over short periods and correlated with growth rates, stress resistance indicators, and gene expression. Hence, when growth rates decline, it should be intervened in by partially altering the water. Through changes in gene expression, more resilient strains to deteriorating water quality can be developed.

## Data Availability

The datasets used and/or analyzed during the current study available from the corresponding author on reasonable request.
